# Overexpression of Sphingosine Kinase-1 and Sphingosine-1-Phosphate Receptor-3 in Severe *Plasmodium falciparum* Malaria with Pulmonary Edema

**DOI:** 10.1155/2020/3932569

**Published:** 2020-02-26

**Authors:** Parnpen Viriyavejakul, Chuchard Punsawad

**Affiliations:** ^1^Department of Tropical Pathology, Faculty of Tropical Medicine, Mahidol University, Bangkok 10400, Thailand; ^2^School of Medicine, Walailak University, Nakhon Si Thammarat 80160, Thailand; ^3^Tropical Medicine Research Unit, Research Institute for Health Sciences, Walailak University, Nakhon Si Thammarat 80160, Thailand

## Abstract

Pulmonary edema (PE) is a major cause of pulmonary manifestations of severe *Plasmodium falciparum* malaria and is usually associated with acute lung injury (ALI) and acute respiratory distress syndrome (ARDS). The sphingosine kinase-1 (SphK-1)/sphingosine-1-phosphate receptor-3 (S1PR-3) pathway has recently been reported to affect the pathogenesis of lung injury, but the expression of these proteins in the lungs of severe *P. falciparum* malaria patients has not been investigated. The cellular expression of SphK-1 and S1PR-3 in lung tissues from autopsied patients with *P. falciparum* malaria was investigated using immunohistochemistry (IHC). Lung tissues from patients who died of severe *P. falciparum* malaria were classified into two groups based on histopathological findings: those with PE (18 patients) and those without PE (non-PE, 19 patients). Ten samples of normal lung tissues were used as the control group. The protein expression levels of SphK-1 and S1PR-3 were significantly upregulated in endothelial cells (ECs), alveolar epithelial cells, and alveolar macrophages (AMs) in the lungs of severe *P. falciparum* malaria patients with PE compared to those in the non-PE and control groups (all *p* < 0.001). In addition, the SphK-1 and S1PR-3 expression levels were significantly positively correlated in pulmonary ECs (*r*_*s*_ = 0.922, *p* < 0.001), alveolar epithelial cells (*r*_*s*_ = 0.995, *p* < 0.001), and AMs (*r*_*s*_ = 0.969, *p* < 0.001). In conclusion, both the SphK-1 and S1PR-3 proteins were overexpressed in the lung tissues of severe *P. falciparum* malaria patients with PE, suggesting that SphK-1 and S1PR-3 mediate the pathogenesis of PE in severe malaria. Targeting the regulation of SphK-1 and/or S1PR-3 may be an approach to treat pulmonary complications in severe *P. falciparum* patients.

## 1. Introduction

Malaria-induced acute lung injury and acute respiratory distress syndrome (ALI/ARDS) are characterized by acute pulmonary inflammation with increased capillary endothelial and alveolar epithelial permeability, leading to interstitial and alveolar edema and hyaline membrane formation [1, 2]. The incidence of pulmonary complications with ARDS in *Plasmodium falciparum* infection ranges from 5% to 25% in adults and up to 29% in pregnant women [2], but such complications may even develop in infections with *P. knowlesi* [2, 3] and *P. vivax* during or after antimalarial treatment [2, 4]. Currently, the pathogenic mechanisms that lead to ALI/ARDS during severe malaria are unclear. The importance of pulmonary complications during severe *P. falciparum* malaria infection highlights the need to further investigate the pathogenesis of these complications. Several features of lung injury in experimental severe malaria have previously been described, such as the increased expression of circulating vascular endothelial growth factor (VEGF) [5] and the accumulation of leukocytes [6], along with the diminished expression of epithelial sodium channels [7] and the apoptosis of alveolar cells [8] in lung tissues. In addition, previous studies revealed that S1P and its receptors, especially S1PR-3, are involved in the vascular integrity disruption in ALI [9–11], and increased S1PR-3 concentrations have been reported to be associated with mortality in intensive care unit patients with sepsis or ALI [11, 12].

The generation of sphingosine-1-phosphate (S1P) is mediated by sphingosine kinases (SphKs), which catalyze the phosphorylation of sphingosine. The function of SphK-1 and SphK-2 is critical in the regulation of S1P generation from sphingosine in mammalian cells [13–15]. In humans, the highest expression levels of SphK-1 are found in the lungs, spleen, and liver, whereas SphK-2 expression is most frequently demonstrated in the liver and heart [16]. These two isoforms of SphK, which participate in specific cellular functions regulated by independent mechanisms, exhibit different kinetic properties and temporal gene expression patterns during development. A previous report demonstrated that intracellular SphK-1 activation is associated with the enhancement of vascular endothelial barrier protection in lipopolysaccharide- (LPS-) mediated pulmonary inflammation *in vivo* [17]. This finding suggested that the intracellular function of SphK-1 is critical in the regulation of inflammatory responses and the severity of lung injury [16]. The mechanisms by which SphK activity is regulated in the lungs of humans with malaria infection are not fully defined. However, our previous work reported that low levels of S1P expression are associated with the severity of malaria and are correlated with the presence of thrombocytopenia and anemia [18]. S1P performs diverse functions, which are mediated in a receptor-dependent manner through G protein-coupled receptors (S1PR-1–S1PR-5) [19]. In mammals, S1P receptors are widely expressed and are believed to play a role in important physiological processes, such as immune cell trafficking, vascular development, vascular tone control, cardiac function, and vascular permeability [13, 20]. A previous study demonstrated that the action of S1P through S1PR-1 receptor contributed to a barrier-protective effect by increasing cortical actin, which promotes adherens junction and focal adhesion complex formation and stabilization [10]. Moreover, in mice with acute ALI induced by the 2009 influenza A H1N1, administration of S1PR-1 receptor agonist together with an antiviral drug provided a maximum protective effect for ALI [21]. On the other hand, S1P could regulate epithelial integrity by disrupting tight junctions through the S1PR-3 receptor expressed on the pulmonary epithelium [22]. Activation through S1PR-3 leads to the release of Weibel-Palade bodies, organelles in endothelial cells that store various proteins involve in inflammation [23]. The S1PR-3 receptor is thought to play a role in endothelial barrier integrity and dysfunction by enhancing RhoGEF recruitment to lipid rafts, and Rho activation leads to cytoskeletal reorganization and decreased cortical actin [10]. The pulmonary endothelial cell (EC) model of acute LPS-induced injury revealed that the expression levels of S1PR-2 and S1PR-3 were further reduced when ECs were treated with a combination of mesenchymal stem cells (MSCs) and S1P [24]. However, the role of the S1PR-2 receptor in endothelial barrier integrity is still controversial [10].

A recent study revealed that the SphK-1 and S1PR-3 expression levels were significantly upregulated in the lung tissues of malaria-infected mice with ALI/ARDS [25]. Since SphK-1 and S1PR-3 have not been previously studied in the lungs of severe *P. falciparum* malaria patients, the cellular expression of the SphK-1/S1PR-3 axis was investigated by immunohistochemical staining. The semiquantitative analysis results for the cellular expression of SphK-1 and S1PR-3 in the lungs of severe *P. falciparum* malaria patients with PE were compared with the results for the non-PE and control groups. In addition, the correlation between the SphK-1 and S1PR-3 expression levels was analyzed.

## 2. Materials and Methods

### 2.1. Lung Tissue Specimens

This study used paraffin-embedded lung tissues from 37 patients with *P. falciparum* malaria. The tissues were stored at the Department of Tropical Pathology, Faculty of Tropical Medicine, Mahidol University, Thailand [8]. The lung tissues were classified into two groups based on the histopathological findings: tissues exhibiting pulmonary edema (PE) (*n* = 18) and tissues that did not exhibit PE (non-PE) (*n* = 19). Normal lung tissues (*n* = 10) from patients without pathological changes in the lung were used as the control group. The mean ages of the patients were 22.58 and 28.55 years in the PE and the non-PE groups, respectively. The mean parasitemia levels were 464,300.84/*μ*l and 218,675.72/*μ*l in the PE and the non-PE groups, respectively [8]. This study was approved by the Ethics Committee of the Faculty of Tropical Medicine, Mahidol University (MUTM 2018-019-01), and the Ethics Committee on Human Rights Related to Research Involving Human Subjects, Walailak University, Thailand (WU-EC-MD-4-041-59). Additional patient consent for this retrospective study was not required.

### 2.2. Immunohistochemical Staining for SphK-1 and S1PR-3

Lung tissues sectioned at a thickness of 4 *μ*m were deparaffinized in xylene and rehydrated through a graded series of alcohols. Sections were inactivated with 3% hydrogen peroxide to quench endogenous peroxidase activity. After the sections were washed, they were incubated with normal goat serum to block nonspecific binding sites, followed by incubation with primary antibodies: rabbit polyclonal antibodies against SphK-1 (dilution 1 : 200; Abcam, UK) or EDG-3 (S1PR-3) (dilution 1 : 200; Cell Signaling Technology, USA). Subsequently, the sections were incubated with secondary antibody and reacted with avidin-biotin complex (ABC) conjugated to horseradish peroxidase (HRP) (Vectastain ABC Kit; Vector Laboratories, USA) according to the manufacturer's instructions. The reactivity of the antigen-antibody complex was visualized by a DAB kit (Vector Laboratories, USA). Finally, the sections were counterstained with Mayer's hematoxylin, dehydrated, and mounted with a coverslip. Negative controls were processed without incubation with the primary antibody and were stained in each run.

### 2.3. Semiquantitative Analysis of Immunohistochemical Staining for SphK-1 and S1PR-3

Ten random microscopic fields at high magnification (400x) were selected to determine the number of cells immunopositive for each target marker. The expression levels of SphK-1 and S1PR-3 were separately examined in three different pulmonary cell types: ECs, alveolar epithelial cells, and alveolar macrophages (AMs). The different cell types in the alveolar walls were morphologically identified as alveolar type I cells, which are thin and flat cells that line almost the entire alveolar surface, whereas alveolar type II cells are cuboidal and often bulging into the alveolus. Alveolar macrophages are pleomorphic in shape and adhere to the internal surface of alveolar septum. Finally, endothelial cells were identified as a thin layer of simple, single-layered squamous cells that are continuous in an interalveolar septum. Finally, the percentage of positively stained cells for each protein marker was calculated by dividing the number of positive cells by the total cell count and multiplying this number by 100 [8]. All evaluations were performed by two independent observers (CP and PV) who were blinded to the patients' groups.

### 2.4. Statistical Analysis

Data are presented as the means ± SEMs. Statistical analysis was performed using IBM SPSS Statistics version 23.0 software (SPSS, IL, USA). The normality of the distribution was tested with a Kolmogorov-Smirnov test. Differences between groups were analyzed by a nonparametric Mann–Whitney *U* test. Spearman's rank correlation coefficient was computed to estimate the direction and strength of correlation between the expression levels of SphK-1 and S1PR-3 in the different cell types. Statistical significance was defined as a *p* value of ≤0.05.

## 3. Results

### 3.1. Immunohistochemical Localization for SphK-1 and S1PR-3

**Figure 1:**
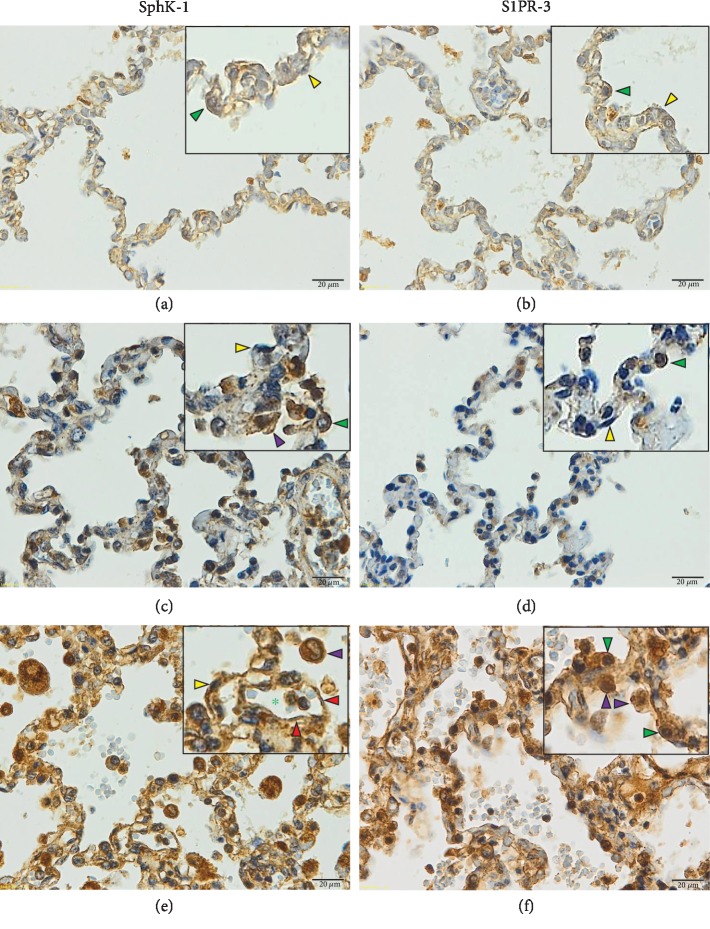
Representative images of immunoperoxidase staining for SphK-1 and S1PR-3 in the lung tissues of severe *P. falciparum* malaria patients. (a, b) Normal lung tissues. (c, d) Lung tissues of severe *P. falciparum* malaria patients without PE. (e, f) Lung tissues of severe *P. falciparum* malaria patients with PE. Green arrowheads indicate alveolar type II cell. Yellow arrowheads indicate alveolar type I cell. Red arrowheads indicate endothelial cells. Purple arrowheads indicate alveolar macrophage. Green asterisks indicate the lumen of the blood vessel. All images were acquired at 400x magnification. Bar = 20 *μ*m.

**Figure 2:**
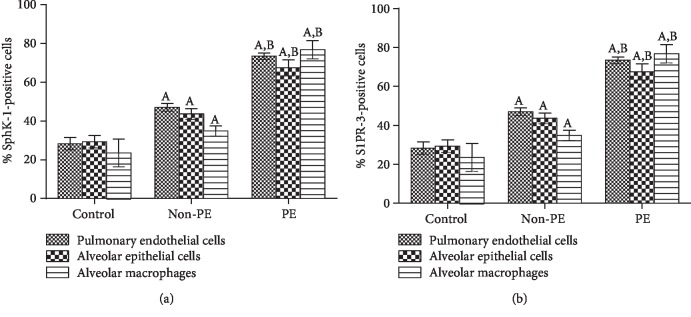
Quantification of SphK-1 and S1PR-3 expression percentages in pulmonary ECs, alveolar epithelial cells, and AMs in the lungs of patients in the non-PE, PE, and control groups. (a) Percentage of cells positive for SphK-1 expression. (b) Percentage of cells positive for S1PR-3 expression. The data are presented as the means ± SEMs. ^A^Significance level of *p* < 0.001 compared with the control group. ^B^Significance level of *p* < 0.001 compared with the non-PE group.

Positive staining for cytoplasmic and nuclear SphK-1 and S1PR-3 was detected in pulmonary ECs, alveolar epithelial cells, and AMs in all groups. In addition, inflammatory cells expressed SphK-1 and S1PR-3. Minimal expression was observed in the control (Figures 1(a) and 1(b)) and non-PE (Figures 1(c) and 1(d)) groups. In contrast, the expression of the SphK-1 and S1PR-3 proteins was strongly upregulated in the lung tissues of patients with PE (Figures 1(e) and 1(f)).The mean percentages of positive SphK-1 and S1PR-3 immunostaining in pulmonary ECs, alveolar epithelial cells, and AMs determined by the semiquantitative analysis are shown in Figure 2. The mean number of SphK-1- and S1PR-3-immunopositive cells in the pulmonary ECs, alveolar epithelial cells, and AMs in the examined lung tissues was significantly higher in the PE group than that in the non-PE and control groups (all *p* < 0.001).

### 3.2. Correlations between the Expression Levels of SphK-1 and S1PR-3

**Figure 3:**
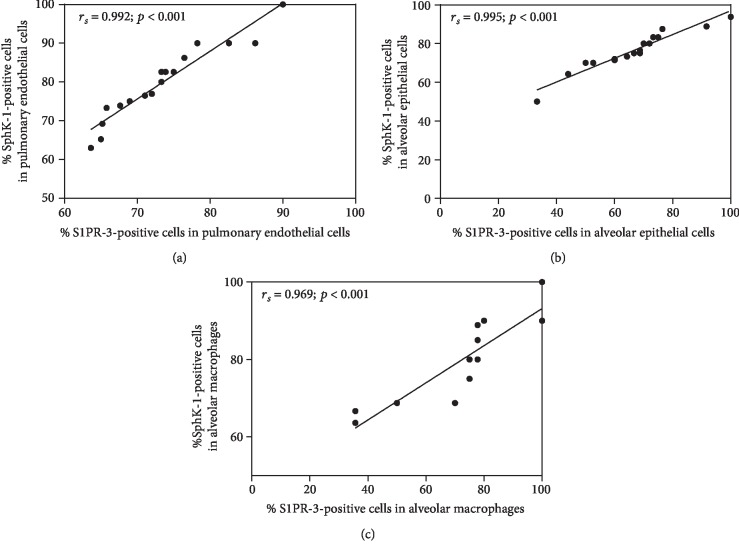
Correlations between the SphK-1 and S1PR-3 expression levels in the lungs of patients with severe *P. falciparum* and PE were assessed by using Spearman's rank correlation test. Positive correlations were found between the SphK-1 and S1PR-3 expression levels in (a) pulmonary ECs, (b) alveolar epithelial cells, and (c) AMs.

The correlations between the expression levels of SphK-1 and S1PR-3 in the lung tissues of the PE group are shown in Figure 3. Significant positive correlations were found between the percentages of cells staining positive for SphK-1 and S1PR-3 in pulmonary ECs (*r*_*s*_ = 0.992; *p* < 0.001) (Figure 3(a)), alveolar epithelial cells (*r*_*s*_ = 0.995; *p* < 0.001) (Figure 3(b)), and AMs (*r*_*s*_ = 0.969; *p* < 0.001) (Figure 3(c)).

## 4. Discussion

In this study, the expression of S1PR-3 was markedly higher in pulmonary ECs, alveolar epithelial cells, and AMs in the lung tissues of patients with severe *P. falciparum* malaria and PE than in the non-PE and control groups. Upregulation of S1PR-3 was reported in lung adenocarcinoma cells [26], astrocytes [27], and a mouse model of cholestasis-induced liver fibrosis [28]. For infectious diseases, elevated expression of S1PR-3 was found in monocytes from patients with bacterial sepsis [29] and in CD4^−^CD8^−^ T cells during the acute phase of *Trypanosoma cruzi* infection [30]. In addition, increased plasma concentrations of S1PR-3 and tyrosine-nitrated S1PR-3 were observed in mice and humans with ALI [11], and both proteins can serve as biomarkers to predict the severity in critically ill ARDS patients [12]. The stimulation of S1PR-3 by S1P results in the activation of the Gq and G12/13 subunits, which leads to an increase in the intracellular calcium levels and facilitates the exocytosis of Weibel-Palade bodies (WPBs) from ECs [23]. WPBs contain von Willebrand factor (VWF) and various other proteins that contribute to inflammation, angiogenesis, and tissue repair [31]. Therefore, S1PR-3 might affect the pathogenesis of PE in malaria.

S1P is a ubiquitous sphingomyelin that is an important regulator of vascular EC permeability. S1P is generally present in plasma and tissues and can increase the resistance of the endothelial barrier [11]. Moreover, S1P also regulates the adhesion of ECs, which is the main mechanism involved in maintaining endothelial barrier integrity and preventing the increased permeability that leads to PE. In this study, there was a decrease in the S1P level, which might contribute to the reduction in the source of S1P in severe malaria, such as red blood cells and platelets; the location of S1P synthesis; and the duration of SphK-1 activation [18]. Furthermore, a reduction in S1P levels may also be due to increased S1P lyase activity [19], which was not determined in this study. In the pulmonary EC model of acute injury by LPS, the expression levels of S1PR-1 and S1PR-3 were significantly increased, but the expression levels of SphK-1 and SphK-2 did not change significantly after LPS induction [24]. We found positive correlations between the expression levels of SphK-1 and S1PR-3 in pulmonary ECs, alveolar epithelial cells, and AMs in the lungs of patients with PE. Moreover, our findings suggest that PE in severe *P. falciparum* malaria evolves through a complex pathophysiological process; thus, treatment targeting a single mechanism may not be optimally efficacious. Therefore, the results of the current study indicate that SphK-1 and S1PR-3 could be therapeutic targets to manage PE in patients with severe *P. falciparum* malaria.

In addition, the current study demonstrated that SphK-1 expression is substantially increased in pulmonary ECs, alveolar epithelial cells, and AMs in the lung tissues of malaria patients with PE. Intracellular SphK-1 activation has been reported to help protect the vascular endothelial barrier during LPS-mediated pulmonary inflammation *in vivo*, independent of the extracellular signaling of its product, S1P. However, S1P is crucial in the regulation of inflammatory responses and the severity of lung injury [17]. A recent study revealed a reduction in serum S1P concentrations in the severe *P. falciparum* malaria group [18]. A previous study in a murine model of LPS-induced lung injury showed that SphK-1 expression increased nearly 8-fold in the first 6 h following LPS treatment and then decreased significantly by 24 h posttreatment [17], suggesting that SphK-1 expression is upregulated in a time-dependent manner. S1P-induced S1PR-3 activation in the alveolar epithelium results in increased permeability via tight junction opening and zonula occludens-1 loss, likely mediated through Rho activation [32]. In contrast, S1PR-1 activation on ECs activates the Rac1 GTPase, inducing the downstream assembly and stabilization of cell-cell junctions with the reorganization of the actin cytoskeleton and vascular endothelial cadherin (VE-cadherin) [22]. In addition, a previous study demonstrated immunopositive staining of SphK-1 in the alveolar cells, foamy macrophages, and ECs of blood vessels in both normal and tumor tissues from the lung [33]. Increased SphK-1 activity was found in the lungs of wild-type mice following PAR-1 activation or LPS challenge, resulting in enhanced S1P generation accompanied by reduced lung edema development [34]. These results suggested that the primary function of SphK-1 is the regulation of endothelial barrier function. In a murine model of LPS-induced lung injury, SphK-1 expression was significantly increased initially but returned to normal levels within 24 h posttreatment, whereas SphK-2 expression exhibited delayed induction but was upregulated within 24 h [17]. Therefore, this study demonstrated that the elevated expression of the SphK-1 protein might be involved in vascular barrier regulation, leading to the development of PE in severe malaria. Further studies should focus on the mechanism of particular cell types with increased expression of SphK1 and S1PR3 during pulmonary edema caused by malaria.

The present study has the following limitations. First, the specimens used in this study were lung tissues from autopsied malaria patients that had been stored for approximately 30 years and were thus unsuitable for determining the gene expression levels of SphK-1 and S1PR-3 via molecular techniques [35]. Second, it was not possible to determine the time course of SphK-1 and S1PR-3 expression during the malaria infection. Further studies on the kinetics of SphK-1 and S1PR-3 expression in samples, such as patient sera, are needed to characterize the time-dependent expression of these proteins. Finally, there might be other unidentified factors involved in the increased expression of S1PR-3, such as bacterial sepsis. Although, no bacterial sepsis was recorded among the enrolled patients, during the treatment period in the past, unrecognized bacterial sepsis may have occurred; thus, this parameter might be one possible confounding factor of the elevated expression of S1PR-3 in our study.

## 5. Conclusions

This study indicates an increase in the protein expression of both SphK-1 and S1PR-3 in pulmonary ECs, alveolar epithelial cells, and AMs in the lungs of severe *P. falciparum* malaria patients with PE. SphK-1 and S1PR-3 may be involved in the regulation of epithelial barrier integrity and endothelial barrier function. Both proteins might play important roles in the pathogenesis of ALI, leading to PE in severe *P. falciparum* malaria. This finding indicated that SphK-1 and S1PR-3 might be therapeutic targets useful in the management of PE during malaria infection.

## Data Availability

The data used to support the findings of this study are included within the article.

## Supplementary Materials

Supplementary Materials Figure S1: negative controls of immunoperoxidase staining for SphK-1 and S1PR-3 in the lung tissues of severe *P. falciparum* malaria patients and the control groups.
