# Supplementary material for: Overexpression of Sphingosine Kinase-1 and Sphingosine-1-Phosphate Receptor-3 in Severe Plasmodium falciparum Malaria with Pulmonary Edema
Source: Biomed Res Int. 2020 Feb 26;2020:3932569. doi: 10.1155/2020/3932569 (PMC7061106; doi:10.1155/2020/3932569)
Supplement: Supplementary Materials — Figure S1: negative controls of immunoperoxidase staining for SphK-1 and S1PR-3 in the lung tissues of severe P. falciparum malaria patients and the control groups. [file 3932569.f1.docx]

**Supplementary Figure:**

**Figure S1 Negative controls of immunoperoxidase staining for SphK-1 and S1PR-3 in the lung tissues of severe P. falciparum malaria patients and the control groups.**

**
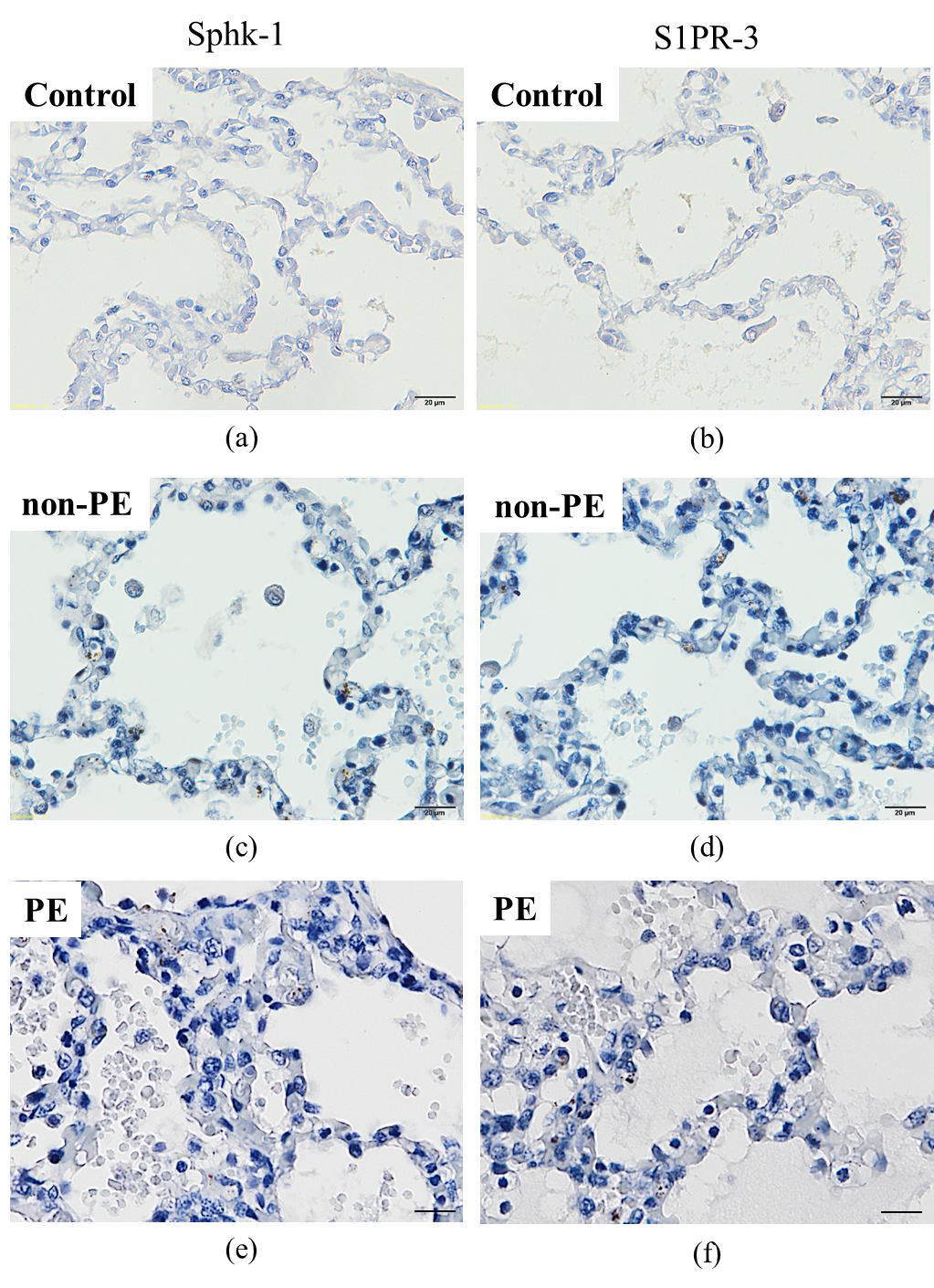
**

**Supplementary Figure Legends:**

Figure S1. Negative controls of immunoperoxidase staining for SphK-1 and S1PR-3 in the lung tissues of severe *P. falciparum* malaria patients. (a, b) Normal lung tissues. (c, d) Lung tissues of severe *P. falciparum* malaria patients without PE. (e, f) Lung tissues of severe *P. falciparum* malaria patients with PE. All images were acquired at 400x magnification. Bar = 20 µm
